# Retrospective analysis of COVID-19 patients developing otherwise rare complications

**DOI:** 10.1007/s44162-023-00006-x

**Published:** 2023-02-08

**Authors:** Kevin Stepanek, Pritha Chitagi

**Affiliations:** grid.414307.5 0000 0004 4691 9995 St. Joseph Mercy Oakland Hospital/Trinity Health Oakland, Pontiac, Michigan, 44405 Woodward Ave, Pontiac, MI 48341 USA

**Keywords:** COVID-19, SARS-CoV-2, Pneumothorax, Pneumomediastinum, Dialysis

## Abstract

**Background:**

Over the last 2 years, it has been felt that there was a disproportionate incidence of complications including pneumothorax, pneumomediastinum, and renal disease necessitating dialysis in patients with COVID-19 as compared to patients without COVID-19.

**Methods:**

In a retrospective cohort, all patients were admitted to St. Joseph Mercy Oakland Hospital in Pontiac, Michigan, between March 2020 and November 2021. The data collected included age, sex, BMI, length of stay, COVID-19 PCR result, diagnosis of pneumothorax, diagnosis of pneumomediastinum, diagnosis of renal failure, orders for dialysis, and orders for mechanical ventilation.

**Results:**

Nine thousand five hundred twenty-two patients are included in this study, with 35.6% (3,392 patients) COVID-19 suspected or confirmed positive and 64.4% (6130 patients) confirmed COVID-19 negative. There were 29 cases of pneumomediastinum and 24 cases of pneumothorax, none of which occurred in intubated patients. The incidence of pneumomediastinum (*p* = 0.001), CODE BLUE (*p* = 0.01), and mechanical ventilation (*p* = 0.001) was significantly higher in the COVID-19 positive/suspected group. There was no significant difference in incidence of pneumothorax (*p* = 0.294). The incidence of dialysis was significantly higher (*p* < 0.0001) in the COVID-19 negative group.

**Conclusions:**

In review of prior literature and proposed mechanisms, we believe that it was possibly the damage that SARS-CoV-2 inflicts upon lung parenchyma that led to the increased incidence of pneumomediastinum. Given our mixed findings of incidences of pneumomediastinum, pneumothorax, and dialysis, our hope is to remain vigilant to uncover further disease associations and/or complications as more COVID-19 case data becomes available.

## Introduction

Throughout the COVID-19 pandemic, there have been a myriad of studies and suggested complications arising in patients infected with this virus in both acute and chronic settings. At the Internal Medicine Residency Program at St. Joseph Mercy Oakland Hospital in Pontiac, Michigan, it was felt that there was a disproportionate incidence of acute complications including pneumothorax, pneumomediastinum, and progression of renal disease necessitating dialysis.

There have been several studies across the globe investigating complications such as this in COVID-19 patients. A 2021 case series from Turkey found 10 patients COVID-19 patients who developed pneumothorax, pneumomediastinum, and pneumothorax as a result of barotrauma (Guven [1]). A 2020 multicenter retrospective case series in the UK reviewed 71 COVID-19 patients and found 11 cases of pneumomediastinum and 60 cases of pneumothoraces, with 14 of those pneumothoraces occurring in nonintubated patients (Martinelli [2]). A retrospective review from Baylor examined 1200 COVID-19 patients and found 9 patients—8 intubated who developed pneumothorax and/or pneumomediastinum (Aayla [3]) A case series from Texas reviewed 5 COVID-19 patients and found 4 cases of pneumothorax and 3 incidences of pneumomediastinum, all of which occurred prior to intubation (Reyes [4]) There is an additional case report from New Jersey that reviewed 1 COVID-19 patient who developed spontaneous pneumomediastinum prior to intubation (Mohan [5]).

The most significant study was an early 2021 multicenter case control in Spain that reported 1.4 million patients in a 61 different emergency departments (Miró [6]). They found 71,904 COVID-19 patients (40 of which developed spontaneous pneumothorax) and 1.3 million non-COVID-19 patients (387 which developed spontaneous pneumothorax). Despite being less than 1% of the cases, they found that spontaneous pneumothorax still occurred in a higher proportion of patients in the COVID-19 group (0.56%) as compared to the non COVID-19 group (0.28%). It was additionally found that the COVID-19 positive patients more frequently had symptoms of dyspnea and chest pain with findings of hypoxia, tachypnea, and leukocytosis.

## Methods

This study was designed as a retrospective cohort of all patients admitted to St. Joseph Mercy Oakland Hospital in Pontiac Michigan between March 2020 and November 2021. At our facility, a patient being admitted must have a COVID-19 PCR performed in the Emergency Department prior to assigning them a floor or ICU bed to avoid cross-contamination. Throughout the pandemic, our facility has had COVID-19 floors and triage areas. Our data was gathered with the help of the Trinity health EPIC data and bioinformatics department and our data was analyzed by a statistician using the SPSS version 25 software for crosstabs, chi-square tests, and *T*-tests. The EMR utilized at our facility has 3 options for COVID-19 status in a patient’s chart—COVID-19 negative, COVID-19 suspected, and COVID-19 positive. The data was sorted into 2 groups—COVID-19 negative patients or COVID-19 positive and COVID-19 suspected patients. The COVID-19 suspected group was grouped together with the confirmed positive group because of high clinical suspicion based on the patient’s presenting illness. Within our EMR, if the COVID-19 test results negative, then the patient’s COVID-19 status in the chart will automatically change from suspected or positive (if tested positive before) to negative. If the patient expires, is discharged, or was transferred to a different facility prior to the test resulting, it would essentially leave the COVID-19 status “stuck” on COVID-19 suspected. In the instance where a patient with a pending COVID-19 test again presented to our facility, the results of that test would then reflect upon their chart. COVID-19 testing at our facility is via the SARS–COV2–RNA qualitative RT-PCR performed on the Abbott Alinity m system.

There was no funding source for this study and no financial disclosures of any kind. The study was approved by the Institutional Review Board at St. Joseph Mercy Oakland Hospital.

## Results

**Figure 1:**
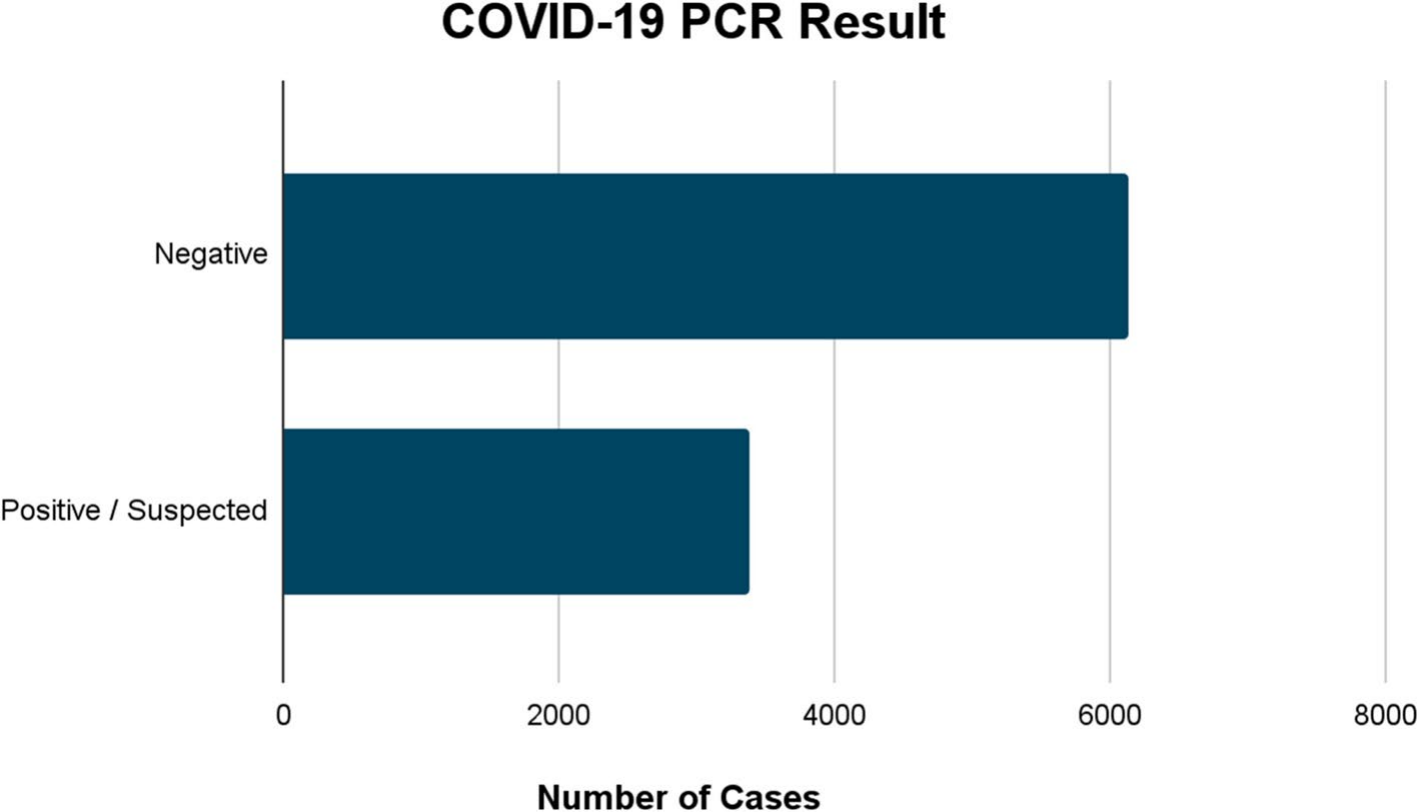
COVID-19 PCR result, divided into positive/suspected or negative

A total of 9,522 patients were admitted to St. Joseph Mercy Oakland Hospital from March 2020 to November 2021 (Fig. 1). Among those, 6130 patients (64.4%) were COVID-19 negative, and 3392 patients (35.6%) were COVID-19 positive or COVID-19 suspected.

There were a total of 29 cases of pneumomediastinum, 24 cases of pneumothorax, and 779 patients on dialysis. All cases of pneumomediastinum and pneumothorax occurred in non-intubated patients. The incidence of pneumomediastinum (Table 1) was significantly higher (*p* = 0.001) in the COVID-19 positive/suspected group (19 cases) as compared to the COVID-19 negative group (10 cases). The incidence of pneumothorax (Table 2) had no statistically significant difference (*p* = 0.294) between the COVID-19 positive/suspected group (11 cases) and the COVID-19 negative group (13 cases). The incidence of dialysis (Table 3) was significantly higher (*p* < 0.001) in the COVID-19 negative group (576 cases) as compared to the COVID-19 positive/suspected group (203 cases).

**Table 1:** Cases of pneumomediastinum

Pneumomediastinum	COVID-19 negative	COVID-19 positive/suspected	Total
No	6120	3373	9493
**Yes**	**10**	**19**	**29**
Total	6130	3392	9522

**Table 2:** Cases of pneumothorax

Pneumothorax	COVID-19 negative	COVID-19 positive/suspected	Total
No	6117	3381	9498
**Yes**	**13**	**11**	**24**
Total	6130	3392	9522

**Table 3:** Cases of renal disease necessitating dialysis

Renal disease necessitating dialysis	COVID-19 negative	COVID-19 positive/suspected	Total
No	5554	3189	8743
**Yes**	**576**	**203**	**779**
Total	6130	3392	9522

**Figure 2:**
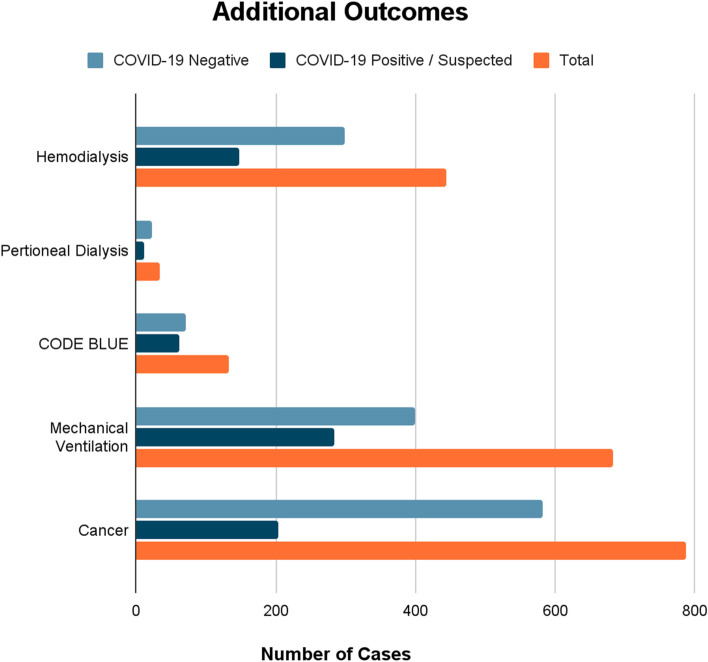
Additional outcomes, grouped by COVID-19 positive/suspected, negative, and total

There was no significant difference for hemodialysis (*p* = 0.265) or peritoneal dialysis (*p* = 1.00) individually between the groups. Additional findings included a significantly higher incidence of CODE BLUE in COVID-19 positive/suspected group (*p* = 0.01). There was a significantly higher incidence of mechanical ventilation in the COVID-19 positive/suspected group (*p* = 0.001). Mechanical ventilation was defined as the presence of orders for ventilator management or utilization of the mechanical ventilation order set in EPIC. Regarding significant secondary outcomes, it was found that the COVID-19 negative group had a higher incidence of cancer patients (*p* < 0.0001). The COVID-19 positive/suspected group had a significantly higher BMI (*p* < 0.0001) and a higher proportion of Hispanic/Latino patients (*p* < 0.0001). For reference, these additional findings are summarized and displayed in Fig. 2.

## Discussion

None of the cases of pneumothorax or pneumomediastinum were present in patients on initial presentation to the hospital. Additionally, none of the cases of pneumothorax or pneumomediastinum found in our study occurred in intubated patients. Therefore, we knew that barotrauma via intubation and mechanical ventilation could not explain these cases of pneumothorax or pneumomediastinum. This prompted further review of proposed mechanisms in COVID-19 patients that lead to the complications of pneumothorax and pneumomediastinum. The case series in Turkey proposed a number of possible explanations for their studied cases of pneumothorax, pneumomediastinum, and hemothorax (Guven [1]). Namely, they had considered SARS-CoV-2 to potentially cause ischemic parenchymal injury, pulmonary fibrosis, low lung compliance, fistulization between parenchyma and pleura, and/or inflammatory exudate. The multicenter retrospective case series in the UK proposed inflammation, consolidation, and necrosis of pulmonary parenchyma that contributed to their studied cases of pneumothorax and pneumomediastinum (Martinelli [2]). The Baylor retrospective review that found a few cases of pneumothorax and pneumomediastinum proposed a mechanism of peripheral pneumocyte damage, akin to SARS-coronavirus 1 and 2 (Aayla [3]). Therefore, this gave us the idea to review additional literature pertaining to complications in older cases of SARS-coronavirus 1 and 2. There was a 2003 prospective study in Hong Kong that looked at SARS pneumonia and found that it was severe alveolar damage leading to alveolar wall rupture (Peiris [7]). Without further understanding of the disease specifics that definitively lead to these complications, for now, we believe it is additional damage to the lung parenchyma inflicted by the SARS-CoV-2 that further predisposes patients to pneumomediastinum. The role of COVID-19 in the development of pneumothorax remains to be seen. That being said, we still recognize the possibility of the idiopathic or alternative pathophysiology leading to the development of these complications—as evidenced by their occurrence in the COVID-19 negative group.

Our results showed that the incidence of dialysis was significantly higher in the COVID-19 negative group (576/5554 patients) as compared to the COVID-19 positive/suspected group (203/3189 patients). We propose that it could be influenced by the poor prognosis typically attributed to patients with COVID-19 who progress to critical illness. In the instance where a COVID-19 patient progressed to necessitating intubation and mechanical ventilation, we have seen the overwhelming respiratory failure precludes the conversation of initiating dialysis. We propose that early goals of care delineation with patients and families can further empower shared decision making and potentially avoid prolonged suffering in patients with poor prognosis. Another avenue that can be worth investigating is whether these complications occur in COVID-19 patients during their first presentation to the hospital or if these are more akin to long term sequelae. We are finding more and more patients testing positive for COVID-19 a second time, and it is certainly possible that any of these complications can still occur on an admission where they test negative for COVID-19 and nevertheless develop a pneumothorax or pneumomediastinum.

Some study limitations we experienced are that different providers use EPIC (our facility’s electronic medical record system) in different ways. Mainly, some physicians use auto-populated problem lists while others manually write diagnoses into their notes. In conjunction with our data analysis team, we attempted to review as many places as possible in every patients’ chart to find any mention of the diagnoses investigated in this study. Additionally, if a patient were to expire, be discharged, or be transferred prior to the result of their COVID-19 PCR test, their COVID-19 status in the chart would remain as “COVID-19 suspected.” In the instance where a patient presented again to our facility, that result would then be appropriately reflected until they were tested again. The data reviewed only included admitted patients and did not review any patients strictly seen in our emergency department.

## Conclusion

In review of significant results, the COVID-19 positive/suspected group had a higher incidence of pneumomediastinum, code blue, and mechanical ventilation. There was no significant difference in the incidence of pneumothorax between the two groups. The COVID-19 negative group had a significantly higher incidence of dialysis. We propose that it is the damage inflicted upon the lung parenchyma that led to an increased incidence of pneumomediastinum. The role of COVID-19 on pneumothorax remains to be seen. A theory for why the incidence of dialysis was higher in the COVID-19 negative group is due to complex medical decision making that incorporates early goals of care and prioritization of an unremitting principal viral infection. Overall, we recognize that further studies are needed as more data becomes available to continue to investigate additional COVID-19 implications and complications.

## Data Availability

Data is attached to this submission
